# Knockdown of RNF2 induces cell cycle arrest and apoptosis in prostate cancer cells through the upregulation of TXNIP

**DOI:** 10.18632/oncotarget.14142

**Published:** 2016-12-24

**Authors:** Ming Wei, Dian Jiao, Donghui Han, Jieheng Wu, Feilong Wei, Guoxu Zheng, Zhangyan Guo, Wenjin Xi, Fa Yang, Pin Xie, Lingling Zhang, An-Gang Yang, He Wang, Weijun Qin, Weihong Wen

**Affiliations:** ^1^ Department of Urology, Tangdu Hospital, Fourth Military Medical University, 710038 Xi’an, China; ^2^ State Key Laboratory of Cancer Biology, Department of Immunology, Fourth Military Medical University, 710032 Xi’an, China; ^3^ Department of Urology, Xijing Hospital, Fourth Military Medical University, 710032 Xi’an, China

**Keywords:** RNF2, TXNIP, prostate cancer, cell cycle, apoptosis

## Abstract

RNF2, also known as RING1b or RING2, is identified as the catalytic subunit of polycomb repressive complex 1 (PRC1), which mediates the mono-ubiquitination of histone H2A. RNF2 has been proved to have oncogenic function in many kinds of cancers, but the function of RNF2 in prostate cancer (PCa) has not been evaluated. Here we show that PCa tissues showed higher RNF2 expression than the benign prostatic hyperplasia (BPH) tissues. Knockdown of RNF2 in PCa cells resulted in cell cycle arrest, increased apoptosis and inhibited cell proliferation, and the growth of RNF2 knockdown PCa xenografts were obviously inhibited in nude mice. Gene microarray analysis was performed and tumor suppressor gene TXNIP was found to be significantly increased in RNF2 knockdown cells. Simultaneously knockdown of RNF2 and TXNIP can partially rescue the arrested cell cycle, increased apoptosis and inhibited cell proliferation in RNF2 single knockdown cells. Furthermore, ChIP assay result showed that RNF2 enriched at the TXNIP promoter, and the enrichment of RNF2 and ubiquitination of H2A in TXNIP promoter was obviously inhibited in RNF2 knockdown cells. In conclusion, our results demonstrate that RNF2 functions as an oncogene in PCa and RNF2 may regulate the progression of PCa through the inhibition of TXNIP.

## INTRODUCTION

Prostate cancer (PCa) is the most common malignancy and the second leading cause of cancer death in men, which causes 913,000 new cases and over 261,000 deaths worldwide each year [1]. Early stage PCa can be cured by surgery or radiation, but advanced PCa is not curable. Generally, systemic androgen ablation is the mainstay of therapy for disseminated PCa, which aims to suppress the androgen receptor (AR) mediated survival signaling. Unfortunately, inhibition of androgen function, together with or without cytotoxic chemotherapy, is only palliative, but not curative. Until now there have no validated molecular targets for PCa. Therefore, the identification of novel therapeutic targets and the development of new strategies for the treatment of PCa are urgently required.

Epigenetic regulation, including DNA methylation, histone modifications and chromatin remodeling, play important roles in the initiation and development of different cancer types, including PCa [2, 3]. Function study of the epigenetic regulators, not only can imply a causative role for these proteins in cancer development but also can provide potential targets for cancer therapy. Several small molecule inhibitors targeting DNA methyltransferases (DNMTs), histone deacetylases (HDACs) and janus kinase 2 (JAK2) have already been granted approval by the US Food and Drug Administration (FDA) for the treatment of certain cancers [4–6]. Thus, the identification of key epigenetic mechanisms in cancer progression may provide new indications for the development of new therapeutic strategies.

Polycomb group (PcG) proteins have been identified as important epigenetic regulators that are involved in embryonic development, tissue homeostasis, stem cell self-renewal and cancer development and progression [7–10]. PcG proteins exist in multimeric complexes, and there have two main complexes in mammals, called polycomb repressive complex 1 (PRC1) and PRC2 respectively. PRC1 is composed of Polycomb (Pc), polyhomeotic (PH), Bmi1, Ring1a and RNF2 (Ring1b), while PRC2 mainly contains EZH2, EED, and SUZ12 [11–13]. Several PcG proteins have been shown to have oncogenic function and have been demonstrated to be highly expressed in certain cancer types, including PCa [14]. EZH2, which is the catalytic subunit of PRC2, has been shown to be overexpressed in metastatic PCa and high EZH2 level is correlated with poor prognosis [15]. The oncogenic function of EZH2 in castration-resistant prostate cancer indicated that it can be used as a therapeutic target [16, 17]. Bmi1, which is component of PRC1, has also been shown to have higher expression than normal prostatic epithelial cells and PIN, and overexpression of Bmi1 formed significantly larger tumors in NOD/SCID mice [18].

RNF2 is the catalytic subunit of PRC1. RNF2 has also been found to be highly expressed in large series of cancer types including breast cancer, ovarian cancer, pancreatic cancer, bladder cancer, melanoma, lymphoma, et al. And high expression of RNF2 is positively correlated with tumor progression and shortened survival, thus RNF2 is considered to be a prognostic biomarker and potential therapeutic target for these cancer types [19–22]. RNF2 has also been shown to be highly expressed in PCa compared with normal tissues [19], yet whether RNF2 plays an oncogenic function in PCa and the potential mechanism is not clear.

The goal of this study is to investigate the function of RNF2 in PCa and to explore the potential mechanism. In this paper, we showed that RNF2 is highly expressed in PCa tissues than the benign prostatic hyperplasia (BPH) tissues, and knockdown of RNF2 in PCa cells resulted in cell cycle arrest, increased apoptosis and inhibited cell proliferation. The oncogenic function of RNF2 was also confirmed in PCa xenograft nude mice. Furthermore, we identified that thoredoxin interacting protein (TXNIP) is a functional target of RNF2. RNF2 is enriched in the promoter of TXNIP, and RNF2 may regulate TXNIP expression through the ubiquitination on histone H2A.

## RESULTS

### PCa tumor tissues showed higher RNF2 expression than BPH tissues

**Figure 1:**
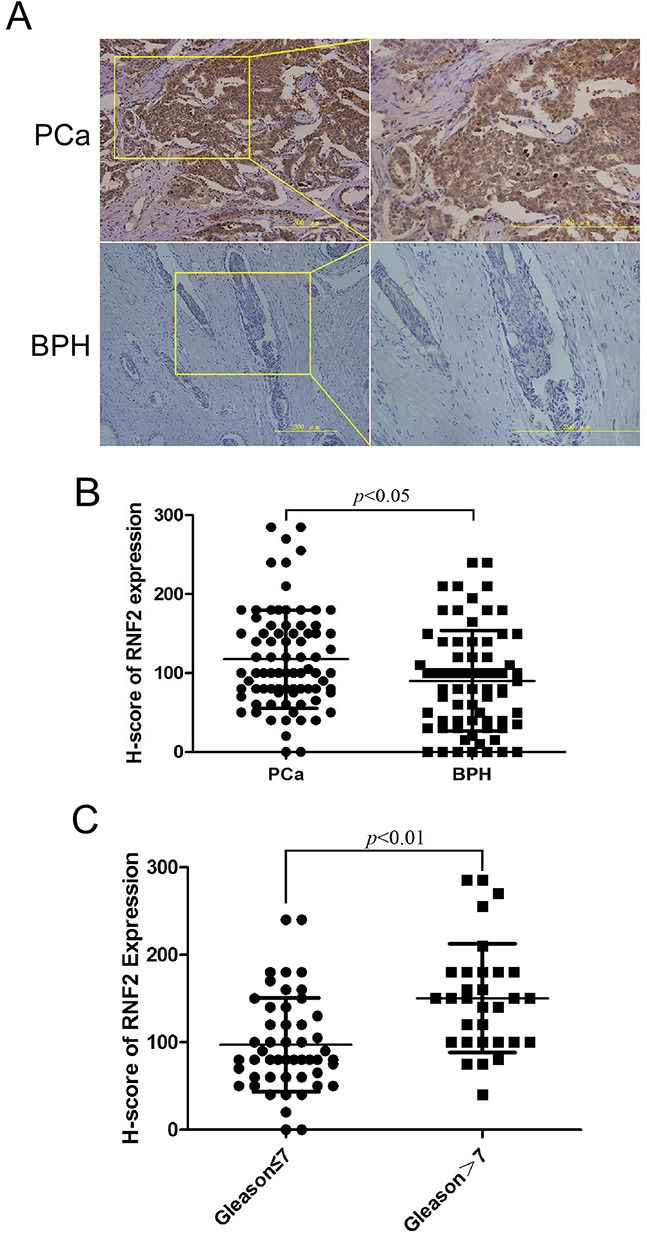
PCa tumor tissues showed higher RNF2 expression than BPH tissues **A**. Representative IHC staining to show RNF2 expression in PCa and BPH tissues. The brown color indicates the positive expression of RNF2. Right pictures are the magnified view of the yellow box in the left. Scale bar: 200 μm. **B**. Statistical analysis of H-score of RNF2 expression in PCa and BPH tissues. **C**. Statistical analysis of H-score of RNF2 expression in low and high grade PCa.

To evaluate the expression status of RNF2 in PCa, IHC staining was performed on two tissue microarray sections including 81 PCa tissues and 71 BPH tissues. Results showed that PCa tissues have obviously higher RNF2 expression than the BPH tissues, with the H-Score of 117.53±62.34 and 90.14±63.66, respectively (*p*<0.05, Figure 1A, 1B). The RNF2 expression level was also analyzed with PCa tumor grade, and results showed that the RNF2 expression was significantly higher in high grade PCa(Gleason>7) (H-Score: 150.5±62.03) than low grade PCa(Gleason≤7) (H-Score: 97.10±53.63) (Figure 1C). These results indicate that PCa tissues have higher RNF2 expression than BPH tissues, and the RNF2 level is positively correlated with tumor grade, indicating that RNF2 may have an oncogenic function in PCa.

### Knockdown of RNF2 resulted in cell cycle arrest and apoptosis in DU145 and LNCaP cells

**Figure 2:**
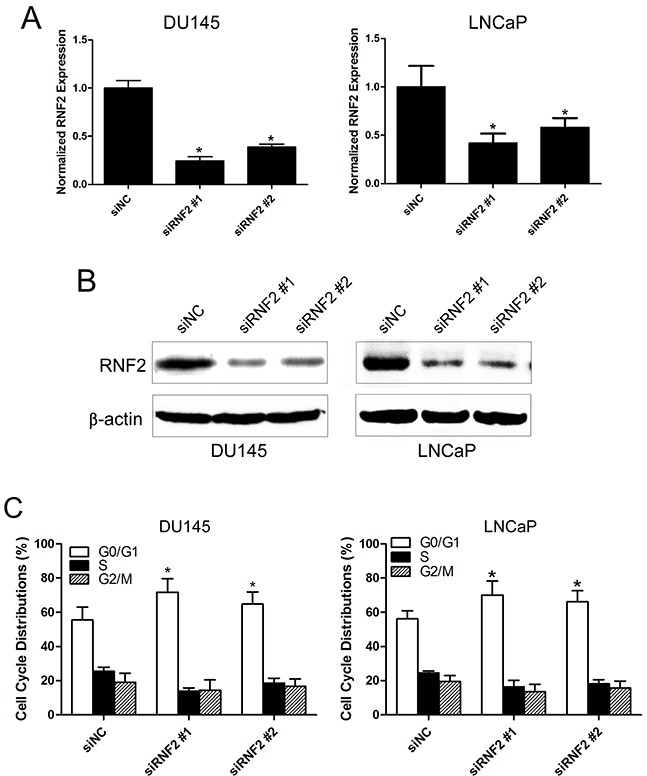

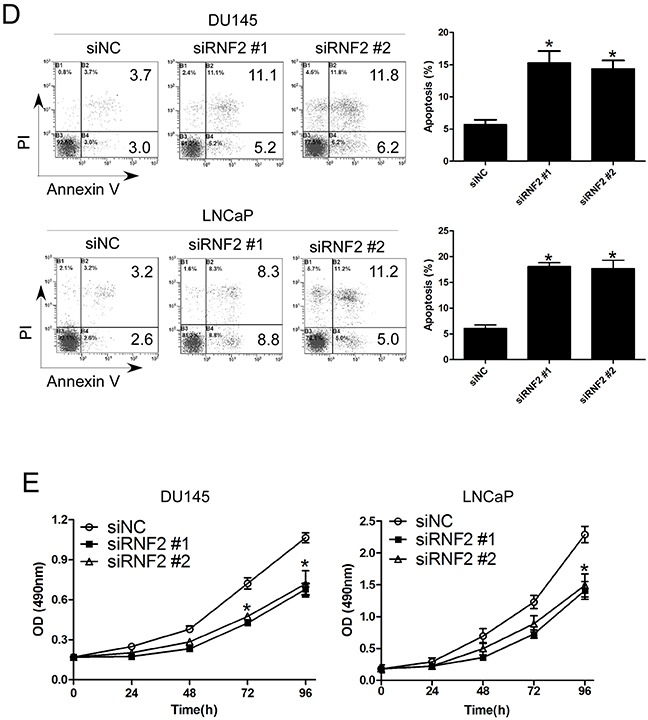
Knockdown of RNF2 resulted in cell cycle arrest and apoptosis in DU145 and LNCaP cells **A**. RT-qPCR analysis to show the knockdown efficiency of RNF2 in DU145 and LNCaP cells transfected with siRNA. *p<0.05 versus siNC. **B**. Western blot analysis to show the knockdown efficiency of RNF2 in DU145 and LNCaP cells transfected with siRNA. **C**. Cell cycle analysis of the RNF2 knockdown and control DU145 and LNCaP cells. Data are shown as the mean ± S.D. from three independent experiments. *p<0.05 versus siNC. **D**. Apoptosis analysis of the RNF2 knockdown and control DU145 and LNCaP cells. Data are representative of three independent experiments. *p<0.05 versus siNC. **E**. MTT assay to show the cell proliferation of RNF2 knockdown and control DU145 and LNCaP cells. Data are shown as mean ± S.D. from three independent experiments. *p<0.05 versus siNC.

To evaluate the biological function of RNF2 in PCa, the expression of RNF2 was knocked down in DU145 and LNCaP cells by siRNAs. The knockdown efficiency was confirmed by RT-qPCR and western blot (Figure 2A, 2B). Cell cycle and apoptosis were analyzed by flow cytometry, and results showed that obvious G1 phase arrest and obvious apoptosis were induced in RNF2 knockdown cells, compared with the control cells (Figure 2C, 2D). The cell proliferation was evaluated using MTT assay, and results show that the proliferation of RNF2 knockdown cells were dramatically inhibited compared with the control cells (Figure 2E). These results indicate that RNF2 plays an important role in the regulation of cell cycle and apoptosis in PCa cells.

### Knockdown of RNF2 inhibited the growth of PCa xenograft in nude mice

**Figure 3:**
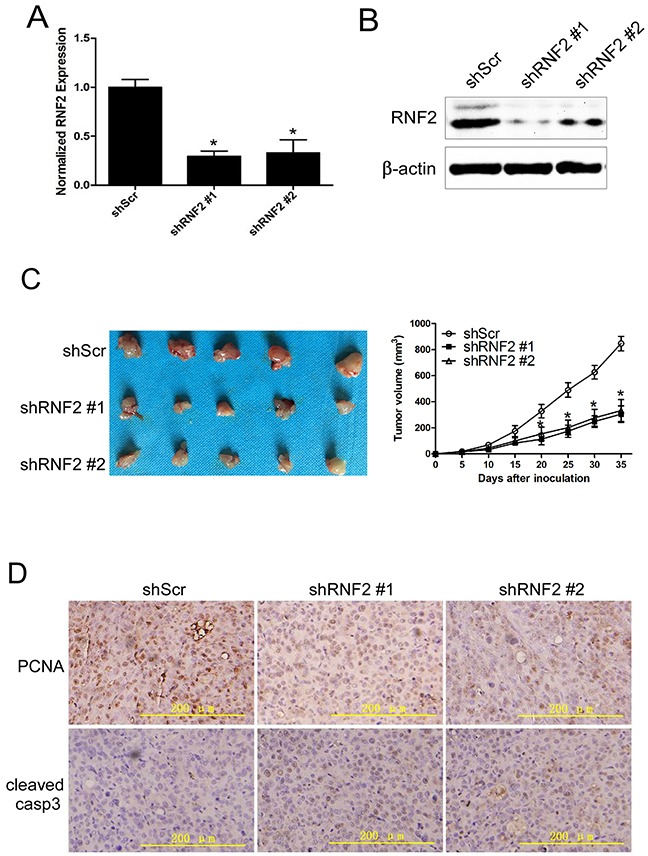
Knockdown of RNF2 inhibited the growth of PCa xenograft in nude mice **A**. RT-qPCR analysis to show the knockdown efficiency of RNF2 in DU145 cells by shRNA expressing lentivirus. *p<0.05 versus shScr. **B**. Western blot analysis to show knockdown efficiency of RNF2 in DU145 cells by shRNA expressing lentivirus. *p<0.05 versus shScr. **C**. Tumor growth curve of the RNF2 knockdown and control DU145 xenografts *in vivo*. Data are shown as the mean ± S.D., *p<0.05 versus shScr. **D**. IHC staining to show the level of PCNA and cleaved caspase-3 in RNF2 knockdown and control DU145 xenograft tumor tissues. The brown color indicates the positive expression of PCNA and cleaved caspase-3. Scale bar: 200 μm.

To evaluate whether knockdown of RNF2 can inhibit PCa tumor growth *in vivo*, we knocked down RNF2 expression using shRNA expressing lentivirus in DU145 cells. The knockdown efficiency of shRNA was confirmed by RT-qPCR and western blot (Figure 3A, 3B). Cell cycle, apoptosis and cell proliferation were also evaluated in lentivirus infected DU145 cells, and shRNF2 lentivirus infected cells also showed obviously G1 arrest, increased apoptosis and inhibited cell proliferation (Supplementary Figure S1A, S1B and S1C). Then, the RNF2 knockdown (shRNF2 #1 and shRNF2 #2) and control DU145 cells (shScr) were inoculated into athymic nude mice. Tumor growth was observed from day 0 to day 35 after inoculation. Results show that the tumor growth in RNF2 knockdown group (shRNF2 #1 and shRNF2 #2) was dramatically inhibited compared with the control (shScr) group, with the final tumor volume of 304.33±63.72 mm^3^, 331.93±84.42 mm^3^ and 846.80±55.29 mm^3^, respectively (*p*<0.05, Figure 3C). To evaluate the difference of the cell proliferation and apoptosis in RNF2 knockdown and control xenograft tumor tissues, IHC staining were performed to detect the level of proliferating cell nuclear antigen (PCNA) and cleaved caspase-3. Results show that the RNF2 knockdown tumor tissue had decreased PCNA level while increased cleaved caspase-3 level than the control tissues (Figure 3D). These results indicate that knockdown of RNF2 can effectively inhibit tumor growth in nude mice through inhibiting cell proliferation and promoting apoptosis. Thus, our data demonstrate that RNF2 has an oncogenic function in PCa.

### TXNIP expression was increased in RNF2 knockdown PCa cells

**Figure 4:**
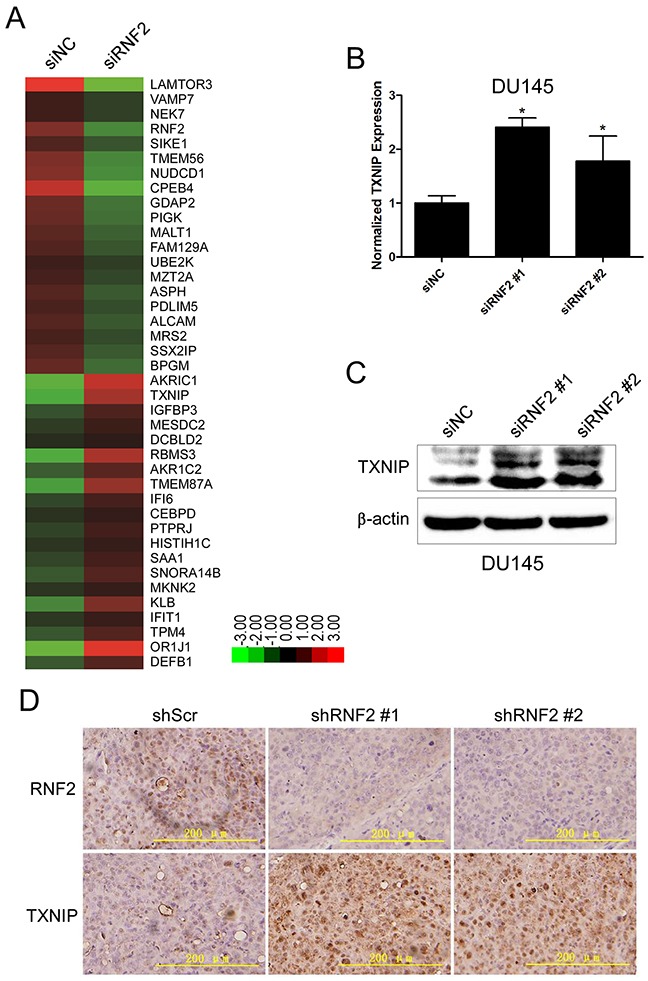
TXNIP expression was increased in RNF2 knockdown DU145 cells **A**. Heat map of the most differentially expressed genes in siNC and siRNF2 cells by microarray analyses (Red: upregulated; Green: downregulated). **B**. RT-qPCR analysis to show the increased TXNIP mRNA level in RNF2 knockdown DU145 cells. *p<0.05 versus siNC. **C**. Western blot analysis to show the increased TXNIP protein level in RNF2 knockdown DU145 cells. *p<0.05 versus siNC. **D**. Representative IHC staining to show the increased TXNIP expression in RNF2 knockdown DU145 xenografts. The brown color indicates the positive expression of RNF2 and TXNIP. Scale bar: 200 μm.

To explore the mechanism how RNF2 regulates cell proliferation and apoptosis in PCa, we performed microarray analyses using the mRNA from RNF2 knockdown and control DU145 cells. Using a 1.5 fold cutoff, we found that 632 genes were differentially expressed in RNF2 knockdown cells. Of these genes, 144 were upregulated and 488 were downregulated. Some of the most significantly changed genes, which participate in the regulation of cell cycle, apoptosis and proliferation, are shown in Figure 4A. Considering the transcriptional repressive function of PcG proteins, we mainly focused on the tumor suppressor genes that were increased in RNF2 knockdown cells, among which, TXNIP is one of the most significantly increased genes. The increased TXNIP expression was confirmed in RNF2 knockdown DU145 and LNCaP cells in both mRNA and protein levels (Figure 4B, 4C, Supplementary Figure S2A, S2B). The expression level of TXNIP was also examined by IHC in RNF2 knockdown DU145 xenograft tumor tissues, and results showed that RNF2 knockdown group had increased TXNIP level than the control group (Figure 4D). Taken together, these results indicate that TXNIP might be one of the functional target genes that are repressed by RNF2.

### Simultaneously knockdown of TXNIP partially rescue the phenotype in RNF2 single knockdown cells

**Figure 5:**
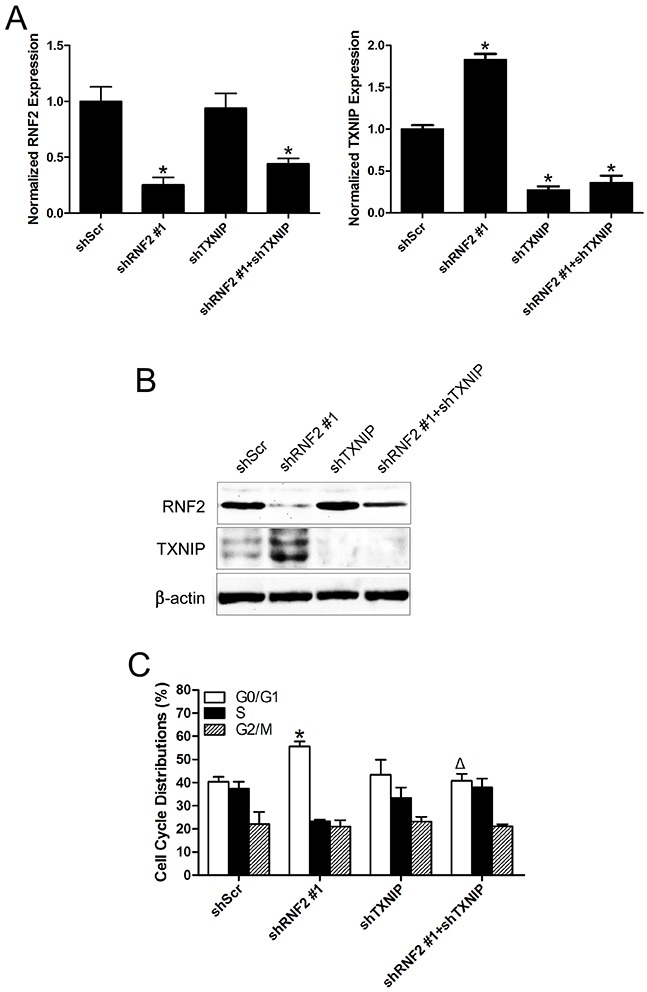

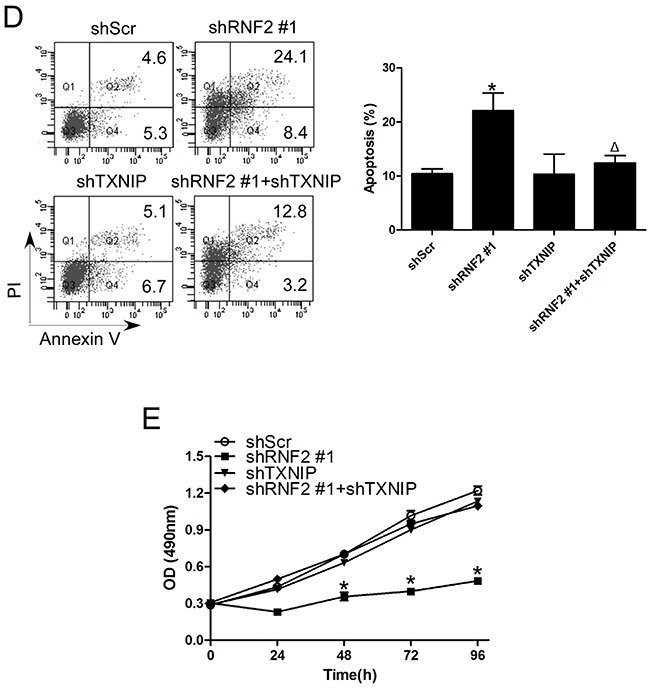
Simultaneously knockdown of TXNIP can partially rescue the phenotype in RNF2 single knockdown DU145 cells **A**. RT-qPCR analysis to show the efficient inhibition of RNF2 and TXNIP mRNA level in DU145 cells infected with shRNA expressing lentivirus. **B**. Western blot analysis to show the efficient knockdown of RNF2 and TXNIP expression in DU145 cells infected with shRNA expressing lentivirus. **C**. Cell cycle analysis in RNF2 and/or TXNIP knockdown and control DU145 cells. Data are shown as the mean ± S.D. from three independent experiments. **D**. Apoptosis analysis in RNF2 and/or TXNIP knockdown and control DU145 cells. Data are representative of three independent experiments. **E**. MTT assay to show the cell proliferation in RNF2 and/or TXNIP knockdown and control DU145 cells. Data are shown as the mean ± S.D. from three independent experiments. *p<0.05 versus shScr, Δp<0.05 versus shRNF2 #1.

To find out whether TXNIP is a functional downstream target of RNF2 in PCa cells, rescue experiments were performed by simultaneously knockdown RNF2 and TXNIP in DU145 and LNCaP cells by either shRNA expressing lentiviruses or siRNA. The knockdown efficiency were confirmed by RT-qPCR and Western blot (Figure 5A, 5B, Supplementary Figure S3A, S3B). Cell cycle and apoptosis were evaluated in RNF2 and/or TXNIP knockdown cells. Results show that simultaneous knockdown of RNF2 and TXNIP can partially rescue the increased G1 phase and apoptosis in RNF2 single knockdown cells (Figure 5C, 5D, Supplementary Figure S3C, S3D). MTT assay was performed to evaluate cell proliferation, and results showed that simultaneously knockdown of RNF2 and TXNIP can rescue the inhibited cell proliferation in RNF2 single knockdown cells (Figure 5E, Supplementary Figure S3E). These results demonstrate that TXNIP is an important downstream molecule in RNF2 mediated regulation of cell proliferation, cell cycle and apoptosis in PCa cells.

### Knockdown of RNF2 decreased the enrichment of RNF2 and H2AK119Ub at the TXNIP promoter

**Figure 6:**
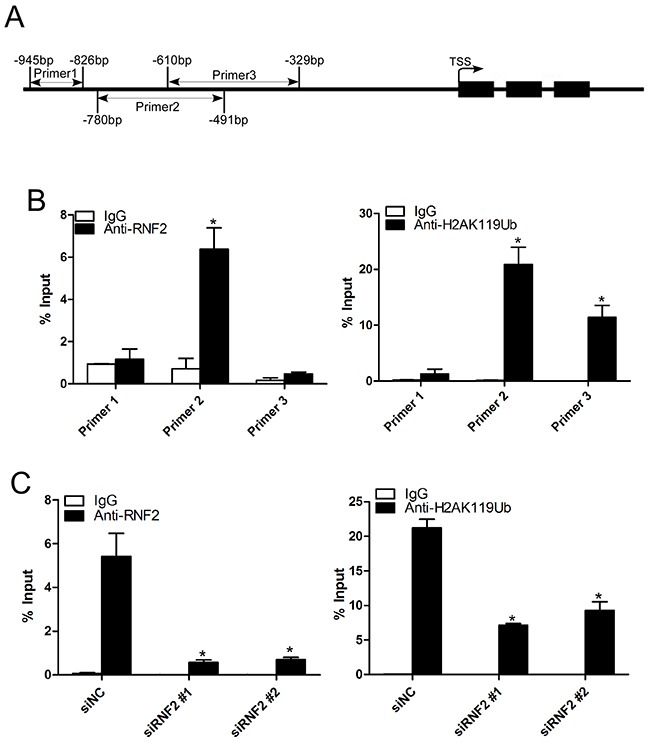
Knockdown of RNF2 decreased the enrichment of RNF2 and H2AK119Ub at the TXNIP promoter **A**. The illustrative picture to show the regions of the PCR products used in ChIP assays. **B**. Quantitative PCR analyses of the ChIP assays using antibodies against RNF2 and H2AK119Ub. Data are shown as mean ± S.D. from three independent experiments. *p<0.05 versus IgG. **C**. Quantitative PCR analyses using primer 3 to show the enrichment of RNF2 and H2AK119Ub at the TXNIP promotor in RNF2 knockdown and control DU145 cells. Data are shown as mean ± S.D. from three independent experiments. *p<0.05 versus siNC.

To further explore the potential mechanism how RNF2 regulates TXNIP expression, we performed ChIP assay to examine whether RNF2 may directly bind at the TXNIP promoter and mediate H2A ubiquitination. Anti-RNF2 and anti-H2AK119Ub antibodies were used for ChIP assay and three pairs of primers were designed to amplify a specific region within 1000 bp upstream of the transcription start site of TXNIP (Figure 6A). Results show that RNF2 and ubiquitinated H2A can be specifically enriched in the TXNIP promoter (Figure 6B). And when RNF2 was knocked down, the enrichment of both RNF2 and ubiquitinated H2A were inhibited (Figure 6C). These results indicate that RNF2 can bind at the promoter of TXNIP and regulates H2A ubiquitination, thus inhibits its expression. We concluded that RNF2 may regulates PCa cell proliferation, apoptosis and cell cycle through direct regulation on TXNIP.

## DISCUSSION

Epigenetic regulation plays important roles not only in normal development but also in different kinds of diseases, including cancer. Among different epigenetic mechanisms, histone post-translational modifications, such as acetylation, methylation and ubiquitination were most intensely studied in recent years [4, 5, 23, 24].

The polycomb group (PcG) proteins are important epigenetic regulators that are involved in the regulation of histone modification. PcG proteins work as transcriptional repressors and play important roles in several developmental and physiological processes. Several PcG proteins such as EZH2 and Bmi1 have been found to play important roles in cancer development and progression, including PCa [25–27]. And certain genes have been identified to be the targets of PcG proteins and the repressed expression of these genes is associated with poor prognosis in PCa [28–30].

RNF2 is the catalytic subunit of PRC1 complex, which is responsible for the mono-ubiquitination of histone H2A. It has been reported that RNF2 has very low expression in normal tissues, but has high expression in certain cancer types. RNF2 has been considered to be a prognostic biomarker and potential therapeutic target for these cancer types [19–22]. In melanoma, RNF2 has been shown to be oncogenic and prometastatic, and RNF2 can promote metastasis through the inhibition of LTBP2, while its oncogenic function does not require it catalytic activity and RNF2 promotes cell proliferation through direct transcriptional upregulation of CCND2 [31]. Recently, it has been reported that RNF2 can regulate p53 stability through either direct ubiquitination or promoting MDM2 mediated ubiquitination in certain cancer types, providing a possible mechanism how RNF2 function as an oncogene [32, 33]. Despite these findings, the function of RNF2 in PCa is still not clear.

In this study, we found that PCa tissues have much higher RNF2 expression than BPH tissues, and RNF2 expression is positively correlated with tumor grade, indicating that RNF2 may have oncogenic function in PCa. We further explored the function of RNF2 in PCa cells using siRNA or shRNA, and found that knockdown of RNF2 resulted in cell cycle arrest, increased apoptosis and inhibited cell proliferation in DU145 and LNCaP cells. In addition, RNF2 knockdown DU145 xenografts showed significantly inhibited tumor growth in nude mice. These results are consistent with other reports, which showed that RNF2 participates in the regulation of cell proliferation, cell cycle and apoptosis [20, 22, 32, 34, 35].

To elucidate the underlying mechanism how RNF2 functions as an oncogene in PCa, we performed microarray analysis using RNA from RNF2 knockdown and control DU145 cells. Plenty of genes were found to be differentially expressed in RNF2 knockdown and control cells. Based on the phenomenon that RNF2 exert an oncogenic function and can suppress gene expression, we mainly focused on several tumor suppressor genes. We identified that the expression of tumor suppressor gene TXNIP was significantly increased in RNF2 knockdown cells. To ensure that TXNIP is a functional downstream target, rescue experiments were performed, and results showed that simultaneously knockdown of TXNIP can partially rescue the inhibited cell proliferation, increased apoptosis and arrested cell cycle in RNF2 single knockdown cells. These results support the conclusion that TXNIP is a functional downstream target of RNF2. Using TCGA data, we also analyzed the TXNIP expression in PCa, and found that the TXNIP expression is significantly decreased in PCa than normal tissues, and the TXNIP expression is inversely correlated with the RNF2 expression in PCa (data not shown).

TXNIP, also known as Vitamin D3 up-regulated protein 1 (VDUP-1) or thioredoxin binding protein 2 (TBP-2), is a key regulator of the redox system, and has been identified as a tumor suppressor [36, 37]. TXNIP is frequently downregulated in many types of human cancers, such as breast cancer, colon cancer, lung cancer, et al [38–42]. TXNIP has been shown to be regulated through epigenetic modification during cancer development, such as DNA methylation and histone deacetylation [43, 44]. TXNIP has also been demonstrated to be the target of PRC2, and the PRC2 inhibitor, 3-Deazaneplanocin A (DZNep), can preferentially induce apoptosis in cancer cells through the induction of TXNIP [45, 46]. The up-regulation of TXNIP expression and subsequent induction of apoptosis have also been shown to be the mechanism of the anti-tumor effect by various agents such as anisomycin, oxidative stress, dexamethasone, PPARγ, calcium influx and potassium ion [47–50].

In our study, to explore the mechanism how RNF2 regulates TXNIP expression, we performed ChIP assays, and found the RNF2 enriches at TXNIP promoter and induce the ubiquitination of H2A at K119, which has been shown to be critical for PRC1-dependent repression of target genes [51]. The RNF2 mediated H2A ubiquitination can be inhibited by Aurora B mediated histone H3S28 phosphorylation, resulting in the transcription of active genes in resting B and T cells [52]. In PCa, how the RNF2 mediated H2A ubiquitination is regulated is not known, and further examinations are also needed to find out whether the RNF2 mediated TXNIP repression is dependent on its catalytic activity on the H2A ubiquitylation.

Taken together, our study demonstrated that RNF2 plays an oncogenic function in PCa, and that TXNIP is an important downstream target of RNF2. Because of the high RNF2 level in PCa tumor tissues and the inhibited cell proliferation and increased apoptosis in RNF2 knockdown cells, we conclude that RNF2 may be used as a new biomarker for diagnosis and new target for the therapy of PCa.

## MATERIALS AND METHODS

### Immunohistochemistry (IHC)

Paraffin-embedded tissue microarray (TMA) sections, including PCa tumor tissues (n=81) and benign prostatic hyperplasia (BPH) tissues (n=71) were subjected to IHC staining to detect the expression level of RNF2. Briefly, slides were deparaffinized in xylene and rehydrated through a graded alcohol series before endogenous peroxidase activity was blocked with 3% H_2_O_2_. Nonspecific protein binding was blocked using pre-immune rabbit serum. The primary antibody for RNF2 (1:1000, Abcam, ab101273) was diluted to recommended concentration and incubated with the sections overnight in a humidified chamber at 4°C. The sections were washed 3 times with phosphate buffer solution (PBS) before HRP conjugated secondary antibody (Elivision^TM^super HRP (Mouse/Rabbit) IHC Kit, KIT-9922, Fuzhou, China) was added and incubated for 30 minutes at room temperature. Visualization was performed using 3,3′-diaminobenzidine (DAB) chromogen for 2 to 3 minutes. The negative control was made by replacing the primary antibody with PBS. Sections were counterstained with hematoxylin. Photos were taken using the Olympus Microscope and DP Controller System (DP70, Japan). RNF2 expression was scored independently by two pathologists blinded to the clinical characteristics of the patients, using H-score method [17]. In brief, the H-score was generated by adding the percentage of strongly stained nuclei (3×), moderately stained nuclei (2×) and weakly stained nuclei (1×), which ranges from 0 to 300. The tumor grades were classified according to the Gleason score. Tumors with the Gleason score ≤7 were low grade, tumors scored 8 to 10 were designated as high grade.

The DU145 xenograft tumor tissues were fixed in 4% formaldehyde immediately after resection and then paraffin-embedded. Tumor slides were subjected to immunohistochemical staining of RNF2 (1:1000, Abcam, ab101273), TXNIP (1:1000, CST, #14715), PCNA (1:200,CST,#13110) and cleaved caspase-3 (1:50, Abcam, ab32042), following the above procedures.

### Cell culture

PCa cell lines (DU145 and LNCaP) were cultured in complete Roswell Park Memorial Institute-1640 (RPMI-1640) medium (HyClone, Logan, UT) supplemented with 10% fetal bovine serum (HyClone, Logan, UT) and 1% penicillin-streptomycin (Invitrogen, Carlsbad, CA). All cells were cultured in a humidified incubator at 37°C in 5% CO_2_.

### RNA interference

For the knockdown of RNF2 or TXNIP using small interfering RNA (siRNA), the following siRNA duplexes were synthesized (Genepharma, Shanghai, China): siRNF2 #1, sense: 5′-GGCUAGAGCUUGAUAAUAATT-3′, anti-sense: 5′-UUAUUAUCAAGCUCUAGCCTT-3′; siRNF2 #2, sense: 5′-CCAGUUCACUGUAUUAAAUTT-3′, anti-sense: 5′-AUUUAAUACAGUGAACUGGTT-3′; siTXNIP, sense: 5′-GAGACCUGGAAACAAAUA UTT-3′, anti-sense: 5′-AUAUUUGUUUCCAGGU CUCTT-3′; siNC, sense: 5′-UUCUCCGAACGUGUCA CGUTT-3′, anti-sense: 5′-ACGUGACACGUUCGGA GAATT-3′. SiNC was used as negative control. The siRNA duplexes (100 nmol/L) were transfected into DU145 or LNCaP cells using Lipofectamine 2000 (Invitrogen, USA) according to the manufacturer's instructions. Cells were harvested 72 hours post-transfection for mRNA or protein analysis.

For the knockdown of RNF2 using shRNA, shRNA-expressing lentivirus vectors were constructed (Genechem, Shanghai, China). The target sequences are: shScr: 5′-TTCTCCGAACGTGTCACGT-3′; shRNF2#1: 5′-AAGTCTACACAGTGAATTA-3′; sh RNF2#2: 5′-ACCTACAAAGGAGCACAAA-3′; shTXNIP: 5′-GACTTATACTGAGGTGGAT-3′. The recombinant lentivirus vectors and packaging vectors were cotransfected into 293T cells for virus package. Supernatants containing lentivirus expressing RNF2-targeting shRNAs, TXNIP-targeting shRNAs and negative control shRNAs were harvested 3 days after transfection. The lentiviruses were then purified by centrifugation, and the titer was confirmed. DU145 and LNCaP cells were infected with the lentivirus at 20 MOI and 5 MOI, respectively.

### Quantitative real-time reverse-transcriptase polymerase chain reaction (RT-qPCR)

Total RNA was extracted using the RNeasy Plus Universal Mini Kit (QIAGEN, Hilden, Germany) according to the manufacturer's protocol. RNA quantity and quality were assessed by NanoDrop 2000 (Thermo, USA). The RNA was reverse transcribed with the Revert Aid^TM^ First Strand cDNA Synthesis Kit (Fermentas, St. Leon-Rot, Germany) according to the manufacturer's instruction. The RT-qPCR was performed using a CFX96^TM^ Real-Time PCR system (BioRad, Valencia, CA) with SYBR Green reagents (#DRR041A; TaKaRa, Japan) according to the manufacturer's instruction. The RT-qPCR analysis was performed in a total volume of 20 μL with the following amplification steps: an initial denaturation step at 95°C for 10 minutes, followed by 40 cycles of denaturation at 95°C for 15 seconds and elongation at 55°C for 30 seconds. Gene expression was normalized to human β-actin and calculated by the 2^−ΔΔCt^ method. Primers used for qPCR were as follows: RNF2, Forward: 5′-AGCACAATAATCAGCAAGCACTC-3′, Reverse: 5′-GCTCCACTACCATTTTCAATCTG-3′; TXNIP: Forward: 5′-CTTGCGGAGTGGCTAAAGTG-3′, Reverse: 5′-TTGAAGGATGTTCCCAGAGG-3′; β-actin: Forward: 5′-TGGCATCCACGAAACTACC-3′, Reverse: 5′-GTGTTGGCGTACAGGTCTT-3′.

### Affymetrix microarray analysis

For the microarray analysis, DU145 cells were transfected with siRNA targeting RNF2. Seventy-two hours after transfection, cells were harvested and total RNA was extracted using the method mentioned above. Total RNA was then subjected to gene expression profiling, using the Affymetrix gene chip array (Biotechnology, Shanghai, China). The gene expression data were log-transformed, median centered and analyzed using the SBC Analysis System (Biotechnology, Shanghai, China).

### Western blot

Whole cell lysates were extracted using RIPA lysis buffer supplemented with protease inhibitor cocktail (Roche, Indianapolis, IN, USA) and quantified by BCA assay. Lysates (30 μg) from each sample were separated by 10% SDS-PAGE and then transferred onto nitrocellulose membranes (Millipore, Bedford, MA). The membranes were blocked in 5% nonfat milk diluted in TBST (Tris Buffered Saline containing 0.05% Tween-20) for 1 hour at room temperature before the addition of the appropriate primary antibody for an overnight incubation at 4°C. Beta-actin was used as loading control. Antibodies used in this study were: anti-RNF2 rabbit monoclonal antibody (1:1000, CST, #5694, Danvers, Massachusetts), anti-TXNIP rabbit monoclonal antibody (1:1000, CST, #14715) and anti-β-actin mouse monoclonal antibody (1:1000, Sigma, A5441). The membranes were then washed with TBST and incubated with the appropriate HRP-conjugated secondary antibody for 1 hour at room temperature. The bands were visualized using a chemiluminescence reagent (New England Nuclear, Boston, MA) and photographed by FluorChem FC2 system (Alpha Innotech, San Leandro, CA).

### MTT assay

Cell proliferation *in vitro* was analyzed using tetrazolium salt 3-(4, 5-dimethylthiazol-2-yl)-2, 5-diphenyltetrazolium bromide (MTT). The DU145 or LNCaP cells were transfected with specific siRNA (siNC, siRNF2 or siTXNIP) for 24 hours or infected with shRNA expressing lentivirus (shScr, shRNF2 or shTXNIP) for 72 hours before MTT analysis. Briefly, 3000 cells of each group were plated in 96-well plates in 100 μL 1640 medium. For analysis: 20 μL of MTT substrate (from a 2.5 mg/ml stock solution in PBS) was added to each well and the plates were incubated for an additional 4 hours at 37°C with 5% CO_2_. The medium was removed and the cells were solubilized in 150 μL dimethylsulfoxide, and colorimetric analysis was performed (wavelength: 492 nm). One plate was analyzed immediately after the cells adhered (approximately 4 hours after plating), and the remaining plates were analyzed every 24 hours for the next four consecutive days.

### Cell cycle and apoptosis analysis by flow cytometry

The DU145 or LNCaP cells were transfected with specific siRNAs (siNC, siRNF2 or siTXNIP) or infected with shRNA expressing lentivirus (shScr, shRNF2 or shTXNIP) for 72 hours before analysis. For cell cycle analysis, cells were harvested and washed with ice-cold PBS before being fixed with 70% ice-cold ethanol. Then, cells were collected by centrifugation and resuspended in PBS containing RNase (100 μg/ml) and Propidium Iodide (PI) (40 μg/ml, BD Bioscience, CA, USA) and incubated at 37°C for 1 hour. Finally, the cell cycle were analyzed by FACS scan flow cytometer (BD, San Jose, CA, USA) and the relative ratios of G1, S and G2 phases were analyzed by FlowJo 2.8 software. For apoptosis analysis, cells were harvested and suspended in PBS at a density of 1×10^6^ cells/mL, and apoptotic cells were analyzed by CYTOMICS FC 500 flow cytometer (Beckman Coulter) after incubation with reagent containing Annexin V-FITC and Propidium Iodide (PI; BD Bioscience, CA, USA) for 15 minutes in darkness at room temperature.

### Xenograft tumor model

The protocol for animal study was approved by the Ethics Committee of the Fourth Military Medical University (FMMU, Xi’an, China). Athymic Balb/c nude mice (from Laboratory Animal Center, FMMU) were housed in individual ventilated cages at (25±1°C) with a 12-hour light/12-hour dark cycle. The use of animals in this study complies with the Guide for the Care and Use of Laboratory Animals (National Institutes of Health publication no. 86–23, revised 1985). Prior to injection, 15 nude mice (5 weeks old, weighing 21.04±1.38 g) were assigned at random into three groups with 5 mice per group (shScr, shRNF2 #1 and shRNF2 #2). Different groups of cells were washed and 5×10^6^ cells were suspended in 0.2 mL RPMI-1640 and then subcutaneously injected into the back, near the right thigh of nude mice. Tumor growth was measured by the tumor diameter with a Vernier caliper every 5 days beginning on the 5th day of injection till the 35^th^ day. Tumor volume was calculated as length×width^2^/2, where the length and width are the longest and shortest axes in millimeters.

### Chromatin immunoprecipitation (ChIP) assay

ChIP assay was performed according to previous report with minor modifications [53]. Briefly, DU145 cells were harvested and cross-linked by 1% formaldehyde for 15 minutes at room temperature. Cells were harvested and resuspended in RIPA lysis buffer in the presence of a protease inhibitor cocktail, then chromatin was sheared by sonication to an average length of 200-1000 base pairs. The sheared chromatin was divided into three groups with equal amount for immunoprecipitation with either anti-RNF2 (CST, #5694), anti-H2AK119Ub (CST, #8240) or control IgG (isotype control)(CST, #2729) with magnetic beads. The immunoprecipitants were eluted, reversed cross-linked, and treated with proteinase K. The purified DNA was subjected to real time quantitative PCR using primers designed based on the region within 1000 bp before the transcription start site of TXNIP. The primers used are as follows: Primer 1: Forward: 5′-TTCCTTTTCCTCCAGAAGCA-3′, Reverse: 5′-TTCTACCTGCAAAGTTGGGG-3′. Primer 2: Forward: 5′-CCTCCTATTTCCGTTCCACA-3′, Reverse: 5′-TACTTTCAGGTTTGGGGCTG-3′. Primer 3: Forward: 5′-CCTCAGAGACGGTGGTGTTT-3′, Reverse: 5′-GGTGTGGACGTTTCTGGTCT-3′.

### Statistical analysis

Statistical analysis was performed using IBM SPSS statistical software (version 20.0). Data were statistically analyzed by Student's t test. P values were determined using 2-sided tests, and P value < 0.05 was considered to be statistically significant. All of the results are presented as the mean ± standard deviation.

## SUPPLEMENTARY FIGURES


