# Supplementary material for: Knockdown of RNF2 induces cell cycle arrest and apoptosis in prostate cancer cells through the upregulation of TXNIP
Source: Oncotarget. 2016 Dec 24;8(3):5323–38. doi: 10.18632/oncotarget.14142 (PMC5354911; doi:10.18632/oncotarget.14142)
Supplement: Supplementary file 1 [file oncotarget-08-5323-s001.pdf]

Knockdown of RNF2 induces cell cycle arrest and apoptosis in prostate cancer cells through the upregulation of TXNIP

SUPPLEMENTARY FIGURES

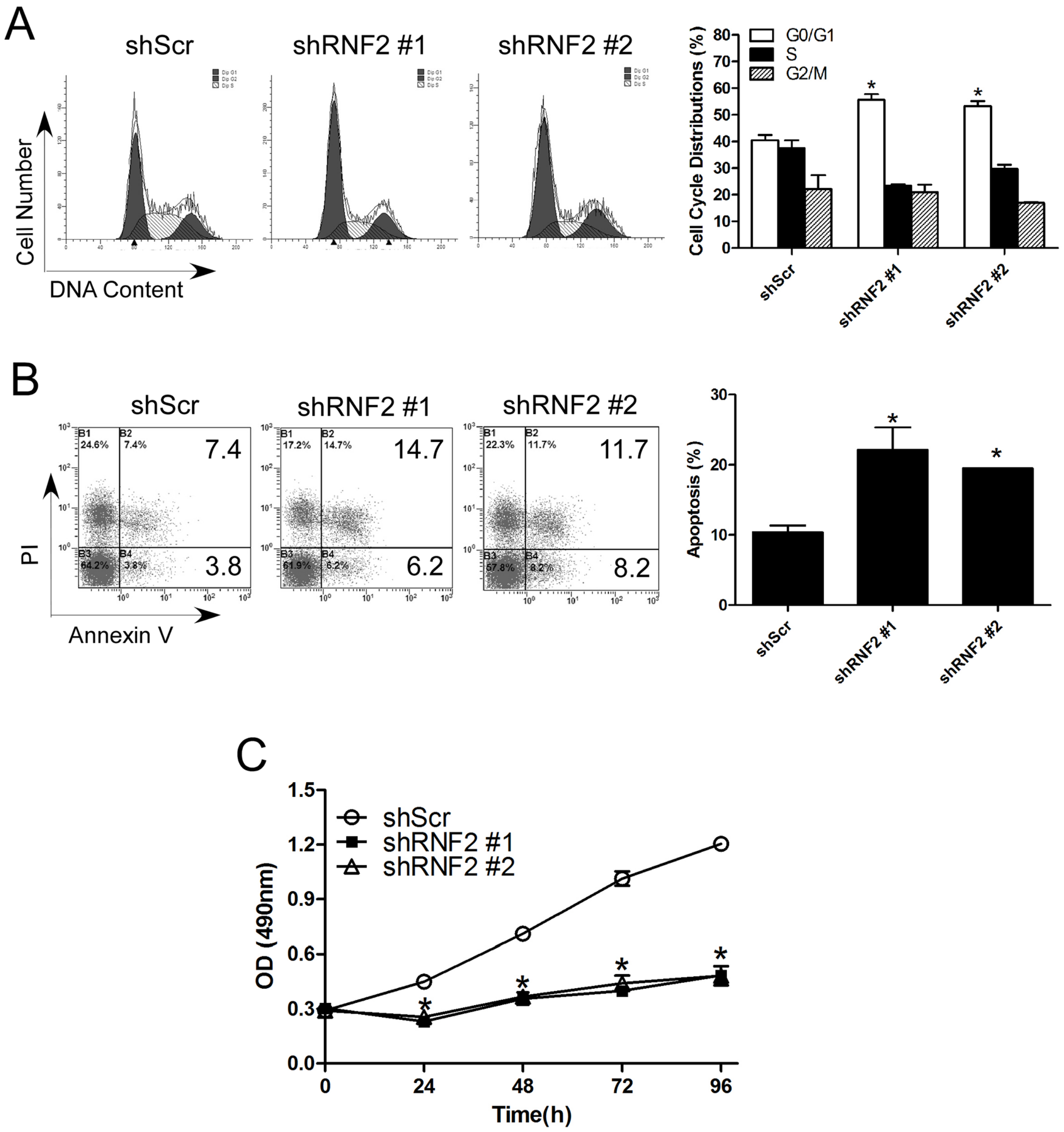

Supplementary Figure S1: Knockdown of RNF2 by shRNA expressing lentivirus resulted in cell cycle arrest and apoptosis in DU145 cells. A. Cell cycle analysis of the RNF2 knockdown and control DU145 cells infected with shRNA expressing lentivirus. Data are shown as the mean ± S.D. from three independent experiments. *p<0.05 versus shScr. **B.** Apoptosis analysis of the RNF2 knockdown and control DU145 cells infected with shRNA expressing lentivirus. Data are representative of three independent experiments. *p<0.05 versus shScr. **C.** MTT assay to show the cell proliferation of RNF2 knockdown and control DU145 cells infected with shRNA expressing lentivirus. Data are shown as mean ± S.D. from one of the 3 independent experiments. *p<0.05 versus shScr.

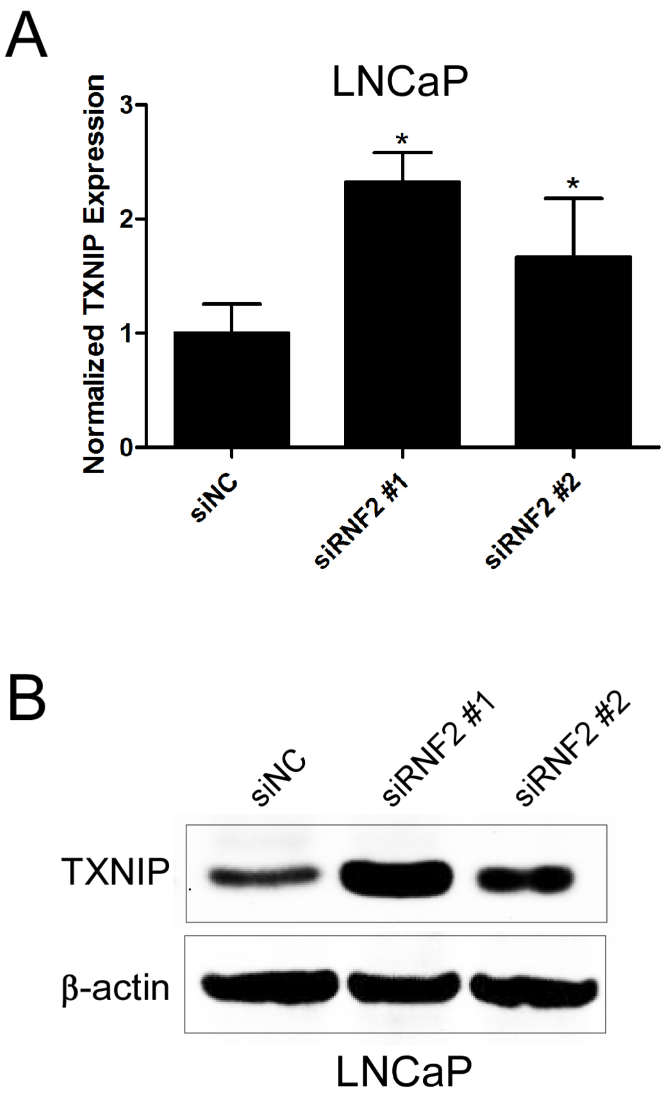

Supplementary Figure S2: The expression of TXNIP increased in RNF2 knockdown LNCaP cells. A. RT-qPCR analysis to show the increased TXNIP mRNA level in RNF2 knockdown LNCaP cells. *$p<0.05$ versus siNC. **B.** Western blot analysis to show the increased TXNIP protein level in RNF2 knockdown LNCaP cells. *$p<0.05$ versus siNC.

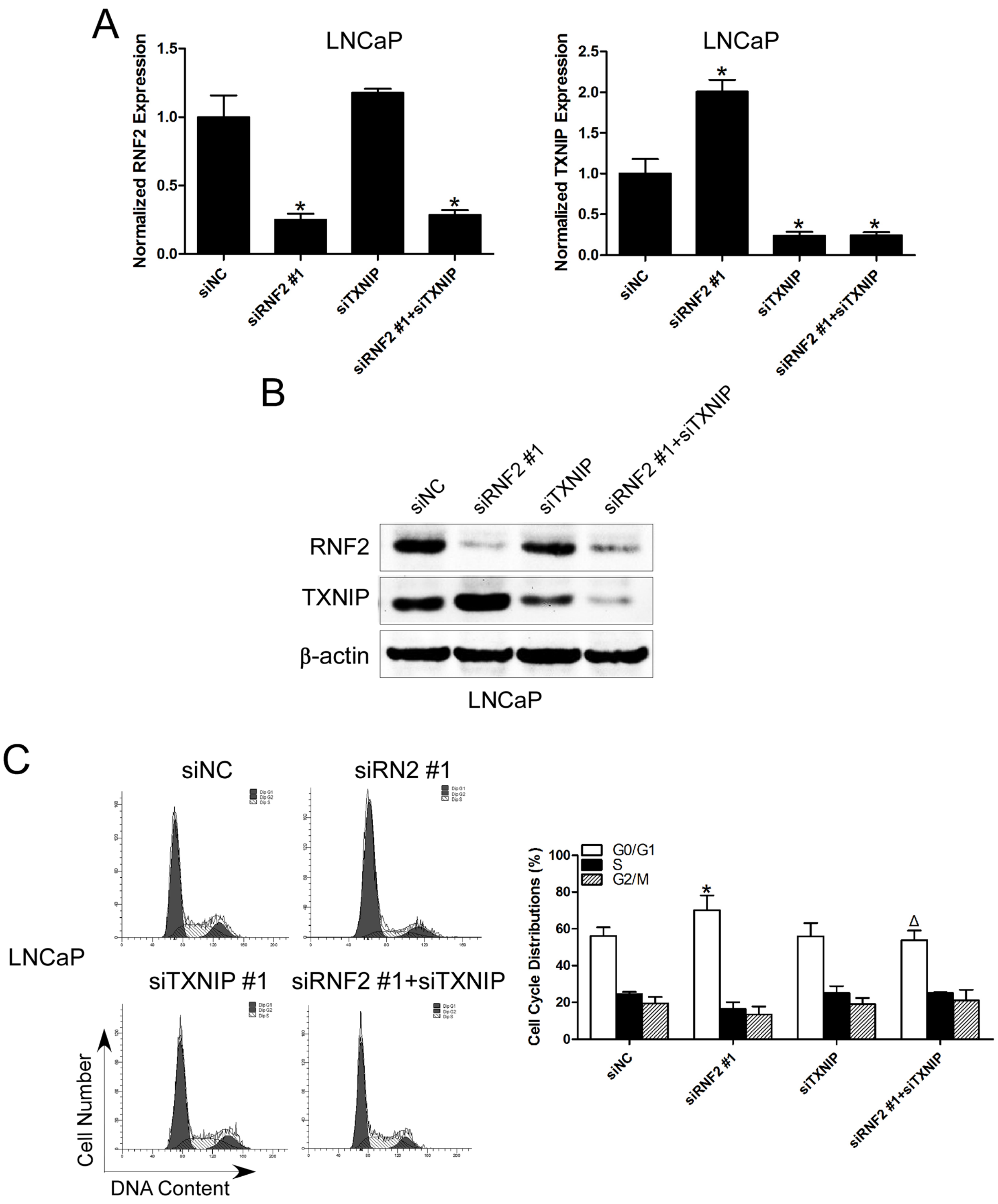

Supplementary Figure S3. Simultaneously knockdown of TXNIP can partially rescue the phenotype in RNF2 single knockdown LNCaP cells. A. RT-qPCR analysis to show the efficient inhibition of RNF2 and TXNIP mRNA level in LNCAP cells by siRNA. *p<0.05 versus siNC. **B.** Western blot analysis to show the efficient knockdown of RNF2 and TXNIP expression in LNCAP cells by siRNA. *p<0.05 versus siNC. **C.** Cell cycle analysis in RNF2 and/or TXNIP knockdown and control LNCAP cells. Data are shown as the mean ± S.D. from three independent experiments. *p<0.05 versus siNC, Δp<0.05 versus siRNF2 #1. (*Continued*)

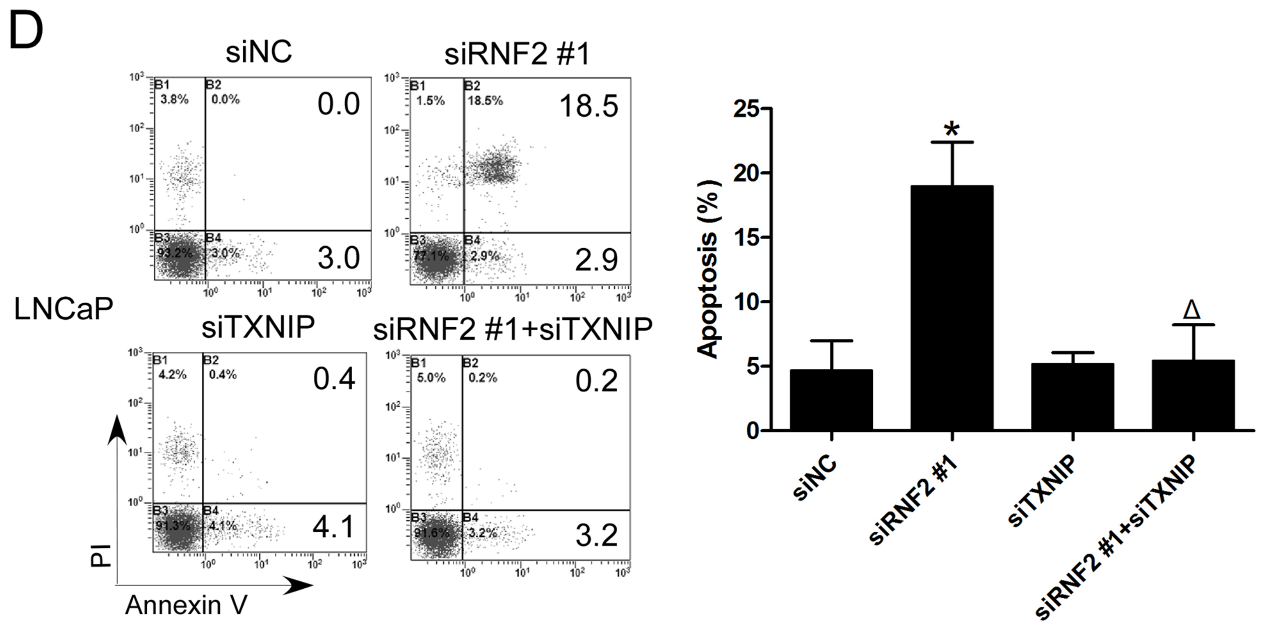

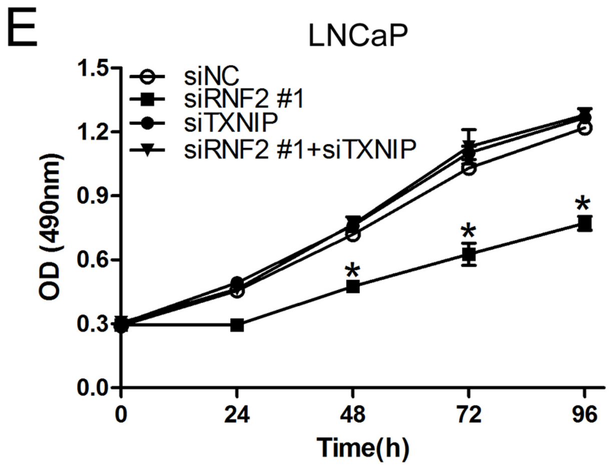

Supplementary Figure S3. (*Continued*) Simultaneously knockdown of TXNIP can partially rescue the phenotype in RNF2 single knockdown LNCaP cells. D. Apoptosis analysis in RNF2 and/or TXNIP knockdown and control LNCAP cells. Data are representative of three independent experiments. *p<0.05 versus siNC, Δp<0.05 versus siRNF2 #1. **E.** MTT assay to show the cell proliferation in RNF2 and/or TXNIP knockdown and control LNCAP cells. Data are shown as the mean ± S.D. from three independent experiments. *p<0.05 versus siNC.
